# Exploring Alcohol’s Effects on Liver Function

**Published:** 1997

**Authors:** Jacquelyn J. Maher

**Affiliations:** Jacquelyn J. Maher, M.D., is associate professor of medicine, University of California, San Francisco, California

**Keywords:** heavy AOD use, alcoholic liver cirrhosis, alcoholic hepatitis, ethanol metabolism, biochemical mechanism, toxic drug effect, inflammation, drug therapy, hereditary factors, gender differences, diet, risk factors, endotoxin, fibrosis, free radicals, etiology, literature review

## Abstract

A large proportion of heavy drinkers develop serious alcoholic liver disease. Susceptibility to alcoholic hepatitis and cirrhosis appears to be influenced by heredity, gender, diet, and co-occurring liver illness. Most alcoholic liver damage is attributed to alcohol metabolism. Liver injury may be caused by direct toxicity of metabolic by-products of alcohol as well as by inflammation induced by these byproducts. Exposure of liver cells to bacterial toxins may contribute to liver disease. Escalating liver injury can lead to fibrosis and, ultimately, to cirrhosis. Increased understanding of the mechanisms of liver injury has led to innovative treatments for alcoholic liver disease, including the use of corticosteroids, antioxidants, antibiotics, and certain polyunsaturated fats.

An association between liver disease and heavy alcohol consumption was recognized more than 200 years ago (Smart and Mann 1992). Long-term heavy alcohol use is the most prevalent single cause of illness and death from liver disease in the United States (National Center for Health Statistics 1994). The liver is particularly susceptible to alcohol-related injury because it is the primary site of alcohol metabolism. As alcohol is broken down in the liver, a number of potentially dangerous by-products are generated, such as acetaldehyde and highly reactive molecules called free radicals. Perhaps more so than alcohol itself, these products contribute to alcohol-induced liver damage.

The liver is one of the largest organs in the body; it has not only considerable reserves but also the ability to regenerate itself. Consequently, symptoms of liver damage may not appear until damage to the organ is quite extensive. Epidemiological studies suggest that a threshold dose of alcohol must be consumed for serious liver injury to become apparent (Mezey et al. 1988). For men, this dose amounts to 600 kilograms (kg) taken chronically over many years, an intake that can be achieved by consuming approximately 72 ounces (oz) of beer, 1 liter of wine, or 8 oz distilled spirits (i.e., 5–6 standard drinks^1^) daily for 20 years. For women, the threshold dose is one-fourth to one-half that amount. In this article, the phrase “heavy drinking” refers to this daily intake.

Heavy long-term alcohol consumption clearly plays a major role in the development of alcohol-related liver damage. Yet, no more than one-half of heavy drinkers develop alcoholic hepatitis or cirrhosis (French et al. 1993). This finding suggests that other factors—heredity, environment, or both—interact to influence the course of liver disease. This article examines some types of liver injury and their potential mechanisms, discusses factors that may place people at increased risk for such injury, and provides a brief description of different treatment approaches.

## Types of Alcohol-Induced Liver Damage

Alcohol-related liver damage can be divided into three categories (French et al. 1993):

- *Fatty liver*. Some degree of fat deposition in the liver occurs in almost all heavy drinkers. It also may occur transiently in nonalcoholics after a single drinking session. Fatty liver is reversible and is not believed to lead to more serious damage.
- *Alcoholic hepatiti*s. This disorder is characterized by widespread inflammation and destruction (i.e., necrosis) of liver tissue. Scar tissue may begin to replace healthy liver tissue, a process called fibrosis. Symptoms of alcoholic hepatitis may include fever, jaundice,^2^ and abdominal pain. The condition can be fatal but may be reversible with abstinence. Alcoholic hepatitis occurs in up to 50 percent of heavy drinkers (National Institute on Alcohol Abuse and Alcoholism [NIAAA] 1993).
- *Alcoholic cirrhosis*. This most advanced form of liver disease is diagnosed in 15 to 30 percent of heavy drinkers. Between 40 and 90 percent of the 26,000 annual deaths from cirrhosis are alcohol related (Dufour et al. 1993). A cirrhotic liver is characterized by extensive fibrosis that stiffens blood vessels and distorts the internal structure of the liver. This structural damage results in severe functional impairment, which may lead secondarily to malfunction of other organs, such as the brain and kidneys. Although alcoholic cirrhosis is usually fatal because of complications (e.g., kidney failure and hypertension in the vein carrying blood to the liver [i.e., the portal vein]), it can stabilize with abstinence.

Traditionally, these three conditions have been considered sequentially related, progressing from fatty liver to alcoholic hepatitis to cirrhosis. However, heavy drinkers may develop alcoholic cirrhosis without first developing hepatitis. Moreover, alcoholic hepatitis may have a sudden onset and a rapid course, causing death before cirrhosis can develop.

## Metabolism of Alcohol

An understanding of alcohol metabolism provides the basis for understanding alcohol-induced liver damage. Most of the alcohol that people drink is metabolized in the liver. The major pathway for alcohol metabolism involves the enzyme alcohol dehydrogenase (ADH). This enzyme converts alcohol to acetaldehyde through a chemical process called oxidation.(For more information on the metabolism of alcohol, see the article by Bode, pp. 76–83.)

Acetaldehyde is highly toxic to the body, even in low concentrations. Normally, however, the enzyme aldehyde dehydrogenase (ALDH) rapidly oxidizes acetaldehyde to acetate. Most of the acetate travels through the bloodstream to other parts of the body, where it can enter other metabolic cycles (Lieber 1994) that produce energy or useful molecules. The usual biological role of both ADH and ALDH is to metabolize vitamin A (i.e., retinol).

The microsomal enzyme oxidizing system (MEOS) is an alternate pathway for alcohol metabolism in the liver. Microsomal enzymes belong to a family of proteins called cytochromes. Some cytochromes, located in a cellular substructure called the endoplasmic reticulum (see figure in glossary, pp. 93–96), detoxify harmful substances that enter the body. The MEOS oxidizes alcohol to acetaldehyde by means of a cytochrome called P450 2E1, or CYP2E1, which is found in the endoplasmic reticulum of liver cells. Normally functioning at a low level, CYP2E1 is stimulated (i.e., induced) to a higher level by the presence of alcohol. Thus, the MEOS becomes increasingly important as alcohol consumption becomes heavier and more chronic.^3^

## Possible Mechanisms Influencing Alcohol-Induced Liver Damage

The mechanisms that influence liver injury are both poorly understood and controversial. Moreover, they interact in complex ways. The following sections briefly discuss aspects of these mechanisms and their interactions.

### Role of Oxygen

Oxygen-related factors that influence alcohol-induced liver damage include the effects of free radicals, antioxidants, and hypoxia.

#### Free Radicals

Much of the direct cell damage that occurs during alcoholic liver disease is believed to be caused by free radicals. Free radicals are highly reactive molecular fragments that frequently contain oxygen. Small quantities of free radicals are produced as normal by-products of various metabolic processes. These fragments are quickly scavenged by natural protective molecules in the cell, called antioxidants (e.g., glutathione and vitamins A and E). However, when free radicals are produced in excess or when antioxidant defenses are impaired, the free radicals may interact destructively with vital cell constituents, potentially causing death of the cell.

One common result of free radical attack is the sequential degradation of cell membranes by a process known as lipid peroxidation. This process may destroy the integrity of the membranes both within and surrounding the cell, seriously compromising cell function (Rubin 1993). Researchers have demonstrated that chronic alcohol consumption induces lipid peroxidation in rats and that the degree of lipid peroxidation is related to the extent of liver injury (Nanji et al. 1994).

When alcohol is metabolized in liver cells by CYP2E1, free radicals are produced. In rats, which are somewhat resistant to alcoholic liver injury, disease can be induced when alcohol is administered together with a high-fat diet (Tsukamoto et al. 1990). The fat increases CYP2E1 activity and simultaneously alters membrane composition, making the membrane more susceptible to peroxidation (Nanji et al. 1994).

#### Antioxidants

As mentioned previously, antioxidants are the cell’s defense against free radicals. Chronic alcohol consumption diminishes the levels of these antioxidants and renders liver cells more susceptible to free radical-induced injury. One important antioxidant that is affected by alcohol is glutathione.

Liver cells contain an abundance of glutathione, especially within structures called mitochondria, where most of each cell’s energy is generated. The key enzymes in mitochondria are certain cytochromes that are integral components of the inner mitochondrial membrane. Like CYP2E1, these cytochromes can produce free radicals—hence the need for antioxidant protection. Glutathione is not synthesized in mitochondria; adequate concentrations of glutathione are maintained there by active transport from the cytoplasm through the mitochondrial membrane. Alcohol interferes with the transport of glutathione through membranes, leading to its depletion from mitochondria. The resulting glutathione deficiency may permit mitochondrial damage and cell death by means of unimpeded lipid peroxidation. Similarly, alcohol has been found to decrease concentrations of vitamins A and E in rat livers, resulting in increased lipid peroxidation and liver injury.

#### Hypoxia (Low Oxygen Concentration)

Alcohol metabolism appears to increase oxygen utilization by liver cells, thereby reducing the availability of oxygen for other important cellular functions (see sidebar). This phenomenon is most important in zone 3 of the liver lobules, which normally is exposed to lower concentrations of oxygen than zone 1 or zone 2 (see sidebar). The tendency of hypoxia to occur in zone 3, together with the fact that free radicals are more likely to be formed in this region, may account for the observation that alcoholic liver damage tends to concentrate in zone 3.

**The Liver—Structure and Function**

The liver is the largest organ of the body, weighing 3.3 pounds. It occupies the upper right—and part of the left—section of the abdomen. Delicate fibrous tissue divides the liver into functional units called lobules, cylindrical structures several millimeters (mm) in length and 0.8 to 2 mm in diameter. The human liver contains 50,000 to 100,000 lobules.

At a microscopic level, each lobule is nourished by blood that enters from the blood vessels at the periphery (see figure). These vessels include small branches of the hepatic artery as well as branches of the portal vein, which brings nutrients and other dissolved substances from the intestine. Blood from these peripheral vessels enters channels called sinusoids, where it bathes all the cells in the lobule before exiting through a vein located in the center of the lobule. The sinusoids are lined in part by Kupffer cells, immune-system cells that attack and engulf bacteria and foreign matter in the blood (see Diehl 1993 for more information).

**Figure:**
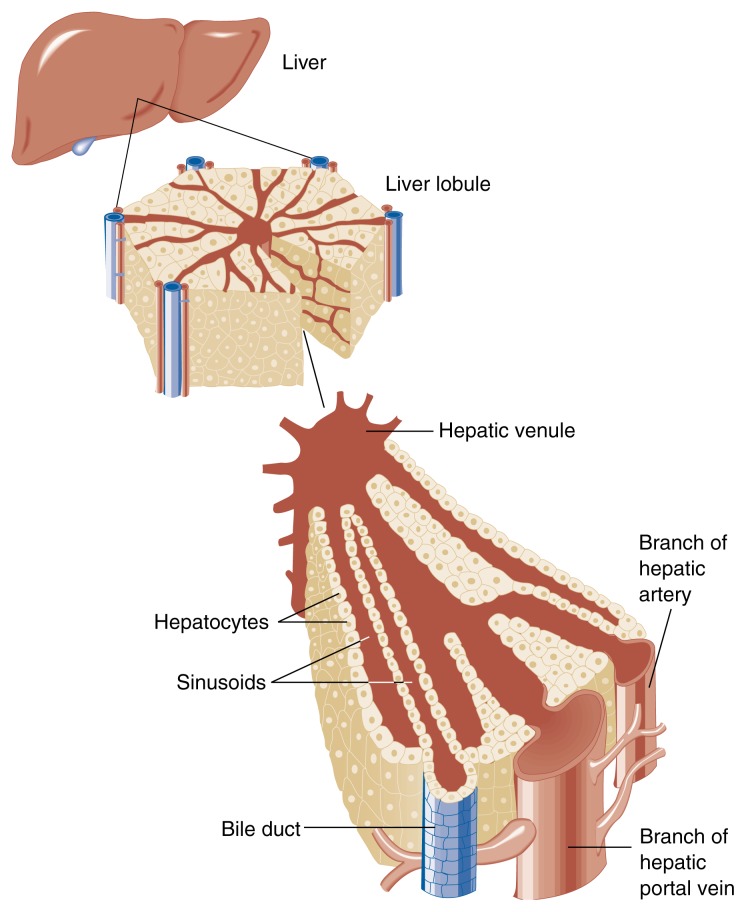
Schematic drawing of liver structure. The liver is divided into functional units called lobules, each organized around a central vein (i.e., the hepatic venule) that returns cleansed blood from the liver to the body.

The unidirectional transport of blood within lobules from the peripheral vessels to the central vein significantly affects liver function and disease. The region adjacent to the peripheral vessels is referred to as zone 1, and the cells surrounding the central vein make up zone 3. Between these zones lies zone 2. Blood flowing through the sinusoids releases dissolved substances, including oxygen, mostly to zone 1 and in the least amount to zone 3. In cirrhotic livers, the relative lack of oxygen (i.e., hypoxia) in zone 3 appears to encourage fibrosis in that zone.

The functions of the liver are essential to life. As the metabolic crossroads of the body, the liver filters circulating blood, removing and destroying toxic substances. It secretes bile into the small intestine to help digest fats and render them soluble for absorption. Nutrients are carried from the small intestine through the portal vein directly to the liver, which then synthesizes cholesterol, metabolizes or stores sugars, processes fats, stores vitamins, and assembles proteins for use within the liver or elsewhere. The liver also converts the products of protein metabolism into urea for excretion by the kidneys. In addition, it regulates blood-clotting mechanisms. The liver can mobilize a chemical and cellular arsenal for self-protection. Fortunately, the liver’s ability to regenerate helps this important organ survive the wear and tear of a lifetime.

ReferenceDiehlAMEffects of alcohol on liver regenerationAlcohol Health & Research World1742792831993

Cells lining the liver sinusoids also may contribute to hypoxia by secreting endothelin, a potent agent that induces narrowing of blood vessels. The resulting narrowing of the sinusoids may decrease the delivery of oxygen-containing blood to zone 3. Patients with cirrhosis also experience increased levels of plasma endothelin, compared with healthy subjects.

### Inflammatory Agents

Inflammation is a localized defensive response to tissue injury. Liver inflammation is the hallmark of alcoholic hepatitis. The inflammatory process begins when liver cells release chemicals that attract specialized white blood cells, or phagocytes, to the damaged tissue. Phagocytes engulf and destroy foreign substances, detoxify bacterial poisons, produce antibodies, and release chemical messengers that attract more phagocytes to the area. Phagocytes arriving from the bloodstream are assisted by a population of phagocytes that remain permanently in the liver, known as Kupffer cells (see figure).

These activities form a highly complex, interrelated, exquisitely regulated network for protection against disease-causing microorganisms and cancer. Under certain circumstances, such as heavy alcohol consumption, the inflammatory process can threaten the body’s own tissues. For example, chronic heavy alcohol consumption can cause an imbalance of certain biological molecules and set in motion other mechanisms leading to tissue damage. Three classes of molecules (i.e., eicosanoids, cytokines, and endotoxins) and two processes (i.e., adduct formation and fibrosis) are the subjects of the following sections.

#### Eicosanoids

Eicosanoids are a family of biological molecules with a wide range of functions. Different eicosanoids affect the liver in different ways: Prostaglandins and prostacyclins can protect liver cells from certain kinds of damage; conversely, thromboxanes cause blood vessels to narrow, which can promote hypoxia or directly cause inflammation or necrosis (Nanji et al. 1993). Another type of eicosanoid, the leukotrienes (e.g., leukotriene B_4_), may cause liver injury by attracting and activating neutrophils, special white blood cells with phagocytic properties. Long-term alcohol consumption alters the balance of eicosanoids in the liver by decreasing the production of cell-protective prostaglandins and prostacyclins and by increasing the synthesis of the harmful eicosanoid thromboxane B_2_ and, possibly, leukotriene B_4_ (Nanji et al. 1993).

#### Cytokines

Cytokines are a family of chemicals produced by various immune system cells, including the Kupffer cells of the liver. As with eicosanoids, the cytokines have many overlapping biological functions, and they can be harmful in the context of long-term heavy alcohol consumption (McClain et al. 1993).

Patients with alcoholic hepatitis frequently have high levels of cytokines in their bloodstream, including tumor necrosis factor alpha (TNF-α) (McClain et al. 1993). TNF-α, produced primarily by Kupffer cells, may cause liver injury directly or indirectly. First, evidence suggests that TNF-α might be directly toxic to liver cells. Second, TNF-α stimulates the liver to produce other cytokines, which attract white blood cells to the liver and stimulate them to release free radicals and toxic enzymes. In experimental animals, TNF-α increases in the liver after 1 month of alcohol administration, timing that coincides with the onset of liver cell necrosis and inflammation.

The mechanisms of increased cytokine production in alcoholic liver disease are not well understood. Chronic exposure to bacterial toxins in the alcoholic may stimulate inflammatory cytokine production (see below) (McClain et al. 1993).

#### Endotoxins

Endotoxins are major molecular constituents of the outer membrane of certain bacteria. Chemically, endotoxins are complex molecules called lipopolysaccharides (LPS’s). These chemicals cause many of the toxic effects of bacterial infection on most organ systems. Endotoxins are present in the intestine because bacteria typically reside there; under normal circumstances, minute amounts of endotoxin may pass through the intestinal lining into the bloodstream. However, alcohol ingestion is believed to increase intestinal permeability to endotoxins, permitting their access to the circulation and subsequently to the liver. Endotoxins are often detectable in the blood of patients with liver disease.

Upon reaching the liver, endotoxins are presumed to act on Kupffer cells, stimulating them to release chemicals that promote inflammation and hypoxia. The ongoing alcohol-induced LPS absorption may provide a continuing stimulus to Kupffer cells that perpetuates inflammation in alcoholic liver disease.

#### Adduct Formation

Highly reactive compounds, such as acetaldehyde and some free radicals, can attach chemically to proteins in the blood and liver. The resulting hybrid molecules are called adducts. The body’s immune system may perceive these protein adducts as foreign and attack them with cellular toxins, white blood cells, and antibodies (Israel et al. 1988). Antibodies directed against protein adducts have been detected in the blood of alcoholics. Adduct formation may contribute to alcoholic liver injury, either by impairing the function of the affected protein or by stimulating an immune mechanism that in turn attacks healthy liver cells.

**Figure:**
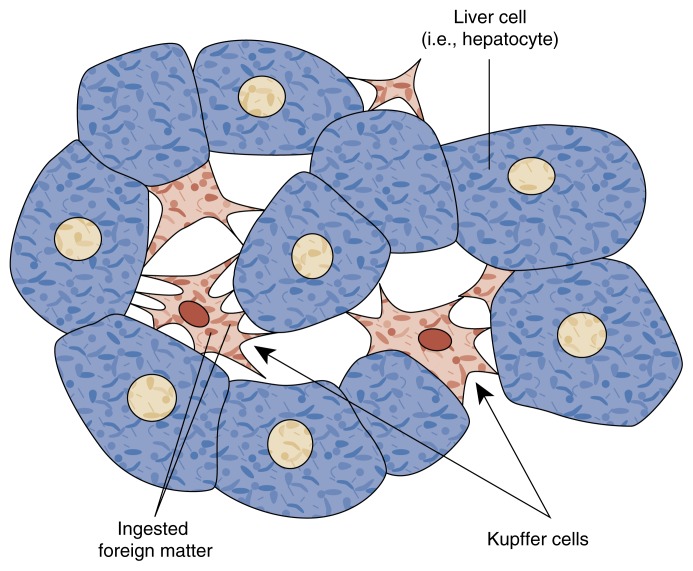
Illustration of Kupffer cells, which line the channels in the liver (i.e., the sinusoids) that contain circulating blood. Kupffer cells filter the blood by ingesting and digesting foreign matter and organisms (i.e., the process of phagocytosis). Dark red areas inside the cells represent ingested foreign particles. SOURCE: Adapted from Guyton, A.C. *Textbook of Medical Physiology*. 4th ed. Philadelphia: W.B. Saunders Co., 1971. p. 109.

#### Fibrosis

Fibrosis is a major mechanism of liver disease because it can lead to irreversible cirrhosis. Long-term alcohol consumption stimulates the liver’s fat-storing (i.e., stellate) cells to produce collagen, the protein that forms scar tissue. The precise stimulus that initiates this process is unknown. Research using liver cells grown in culture indicates that compounds associated with alcoholic liver injury may be involved. For example, acetaldehyde and aldehydeprotein adducts can increase collagen synthesis in vitro, as can the chemical products of lipid peroxidation.

A third potential stimulus to alcoholic liver fibrosis is a cytokine called transforming growth factor beta (TGF-β). In the presence of this cytokine, stellate cells grown in culture begin to synthesize collagen. In rats, chronic alcohol feeding induces TGF-β production by Kupffer cells. TGF-β also is produced by stellate cells themselves; this production can occur in the liver of alcoholics, creating a self-perpetuating cycle of fibrosis.

Kupffer cells may produce compounds other than TGF-β that influence the development of alcoholic liver fibrosis. For example, Matsuoka and colleagues (1990) have shown that Kupffer cells from alcohol-fed rats secrete a growth factor for stellate cells. By increasing the number of stellate cells, Kupffer cells can increase fibrosis indirectly.

## Risk Factors for Liver Disease

Not all alcoholics develop serious liver disease, regardless of the amount of alcohol consumed. Part of the reason for individual susceptibility may be genetic. Researchers are investigating whether variation in alcohol-metabolizing enzymes may play a role in differential susceptibility to alcoholic liver damage.

### Genetic Variations in Enzymes That Metabolize Alcohol

Genes direct the synthesis of all proteins in the body, including enzymes. Genes come in pairs; each half of the pair is called an allele. Gene pairs may or may not comprise identical alleles. Slight differences in a given gene (i.e., polymorphism) among individuals or populations may cause minor variations in the corresponding protein but can lead to drastic differences in enzyme activities, a defective protein, or no protein at all. Polymorphisms in ADH, for example, are responsible for large differences among people in the rates of alcohol metabolism. However, no single ADH polymorphism has been clearly associated with the development of alcoholic liver injury (Lieber 1994).

ALDH variants, however, have been implicated in alcoholic liver disease. Two types of alleles for ALDH exist: ALDH2*1 and ALDH2*2. The allele ALDH2*2 is present in about 50 percent of persons of Chinese or Japanese descent. People whose gene for ALDH consists of paired ALDH2*2 alleles accumulate toxic quantities of acetaldehyde after even moderate drinking, resulting in facial flushing, high blood pressure, increased heart rate, headache, nausea, and vomiting. Consequently, these people generally have an aversion to alcohol. People in whom the ALDH2*2 allele is paired with the ALDH2*1 allele exhibit a less severe toxic response and may drink heavily. However, they develop liver injury with greater frequency and at lower doses than do patients with paired ALDH2*1 alleles.

Researchers also have detected a polymorphism in CYP2E1. People with the rare c2 allele have higher CYP2E1 activity at baseline (i.e., before induction) than do people without this allele. One study demonstrated that in patients with alcoholic liver disease, the c2 allele was more than twice as common as in healthy control subjects or patients with non-alcoholic liver disease (Tsutsumi et al. 1994). The c2 allele has not been found in alcoholics without liver disease, indicating the potential importance of this allele as a risk factor.

### Gender

Women develop cirrhosis at a much lower cumulative dose of alcohol than do men (Mezey et al. 1988). Moreover, women remain at increased risk of disease progression even after abstinence. Two theories have been proposed to explain gender-specific differences in the risk of alcoholic liver disease. The first theory involves gastric ADH. Although it is present at high levels in the liver, ADH occurs in the stomach and intestine as well. Gastric ADH is of potential importance because metabolism of alcohol in the stomach can limit the amount of ingested alcohol that reaches the liver. Women have been reported to have lower levels of gastric ADH activity than do men. In women, therefore, a higher percentage of ingested alcohol reaches the liver than it does in men, possibly predisposing women to earlier onset of alcoholic liver disease.

Differences in the metabolism of fatty acids also may contribute to accelerated alcoholic liver injury in women. Chronic alcohol consumption inhibits the major pathway for fatty acid metabolism, which is located in mitochondria. Inhibition of this pathway in the liver can cause fatty acid accumulation and consequent liver injury. The activation of alternate pathways of fatty acid metabolism can prevent this damage. In rats, however, such activation is less efficient in females than in males, a finding that supports the role of fatty acid toxicity in alcoholic liver damage in women.

### Diet and Nutrition

Dietary factors may facilitate liver injury associated with alcohol metabolism, in part by influencing free radical activity. For example, deficiencies of glutathione or vitamins A or E can decrease the liver’s protection against free radicals. Nutritional factors can induce CYP2E1; certain food components, such as polyunsaturated fats (found in fish, shellfish, and certain vegetables), also can provide excess lipids for peroxidation by free radicals produced by CYP2E1.

Chronic alcohol consumption also promotes absorption of iron from food in the intestine and facilitates storage of iron in the liver. Iron is an important catalyst of free radical production.

### Infection With Hepatitis C Virus

Most people with hepatitis C virus (HCV) have only mild symptoms, such as fatigue; in some cases, however, infection may lead to progressive liver disease, cirrhosis, or liver cancer. Although the transmission of HCV is not well understood, roughly one-half of infected patients have a history of injection drug use (i.e., sharing of dirty needles). Infection with HCV increases the risk of liver injury in alcoholics. Long-term drinking may increase HCV proliferation, which in turn may accelerate HCV-induced damage. Although data on the rate of HCV in the general population are unavailable (about 4,500 cases were reported to the Centers for Disease Control and Prevention [CDC] in 1995 [CDC 1995]), approximately 18 to 25 percent of alcoholics exhibit signs of HCV infection. Among alcoholics with liver damage, more than 40 percent may be infected (Nalpas et al. 1991). Although not all studies have confirmed this high prevalence of HCV in alcoholics with liver disease, most researchers agree that alcoholics infected with HCV develop liver injury at a younger age and at a lower cumulative dose of alcohol than do those without HCV.

### Tobacco and Coffee

Alcoholics who smoke more than one pack of cigarettes per day experience three times the risk of cirrhosis of those who do not smoke. Conversely, alcoholics who consume four or more cups of coffee daily have a fivefold lower incidence of cirrhosis than those who do not drink coffee. The reason for these effects is unknown. Coffee’s effect is not related to caffeine, however, because tea does not have the same effect (Klatsky and Armstrong 1992).

## Treatment of Liver Disease

### Alcoholic Hepatitis

Potential treatments for alcoholic hepatitis are largely directed against inflammation and free radical-induced liver injury.

#### Corticosteroids

Among other functions, corticosteroids act to suppress inflammation. Natural corticosteroids are synthesized from cholesterol by the adrenal glands, which are located above the kidneys. Synthetic corticosteroids, such as prednisolone, are widely used medicinally to suppress inflammation (McClain et al. 1993). Some studies demonstrate that corticosteroid therapy may improve survival in patients with severe alcoholic hepatitis (NIAAA 1993). Other studies have reported significantly improved survival rates only in patients suffering brain-related complications of alcoholic liver disease, however, and not in patients with milder illness (Imperiale and McCullough 1990). (For information on the effects of alcohol on corticosteroid synthesis and release, see the article by Emanuele and Emanuele, pp. 53–64.)

#### Nutrition/Antioxidants

Although it has not been shown to directly improve patient survival, aggressive nutritional support is recommended for all patients with alcoholic liver disease. Given the importance of free radicals as a cause of liver injury, supplementation with antioxidants is a key nutritional goal.

Glutathione depletion has been prevented in alcohol-fed animals by administering *S*-adenosyl-l-methionine (SAM), a precursor of glutathione. Interestingly, SAM’s positive effect seems unrelated to its potential promotion of glutathione synthesis, but apparently derives from its ability to modify the mitochondrial membrane, thereby restoring normal transport of glutathione through that membrane. This effect helps to maintain normal levels of glutathione in the mitochondrion, where it is needed to prevent free radical damage.

Researchers also are investigating vitamins A and E as therapeutic agents for alcoholic liver disease. So far, vitamin E supplements have not significantly prevented or reversed alcoholic liver injury in experiments with laboratory animals. Vitamin A supplementation is not practical because the inherent toxicity of vitamin A severely limits the dose that can be safely administered (Lieber 1994).

#### Antibiotics and Immune System Inhibitors

The role of LPS’s in alcoholic liver disease suggests that using antibiotics to eradicate LPS-containing bacteria from the gut may be helpful. For example, Adachi and colleagues (1995) demonstrated in rats that intestinal sterilization could prevent alcohol-induced liver injury. Along similar lines, researchers have suggested using antibodies or other targeted agents to block the actions of Kupffer cells or cytokines. Antibodies against LPS may be of some use (McClain et al. 1993). In addition, thromboxane inhibitors have prevented necrosis and inflammation in alcohol-treated rats.

### Alcoholic Cirrhosis

Up to a point, liver fibrosis may be reversible with abstinence. Eventually, however, the liver loses its ability to reabsorb scar tissue, and the disease progresses to cirrhosis. Treatment for cirrhosis is directed largely against its symptoms (e.g., bleeding in the esophagus) and complications (e.g., portal vein hypertension).

#### Liver Transplantation

For patients becoming terminally ill, liver transplantation is the only effective treatment. In alcoholic cirrhotic patients, liver transplantation^4^ has demonstrated both success and survival rates equal to those for nonalcoholic subjects (Kumar et al. 1990; Van Thiel et al. 1991).

#### Polyunsaturated Lecithin

Lieber and colleagues (1994) demonstrated that a mixture of fatty substances called polyunsaturated lecithin (PUL) dramatically reduced the incidence of cirrhosis in baboons fed alcohol for several years. In some cases, removal of PUL from the diet led to the development of cirrhosis, perhaps in an accelerated manner (Schenker and Halff 1993). PUL presumably exerts its beneficial effect by promoting the degradation of collagen, thereby inhibiting fibrosis. PUL also may help stabilize membranes and encourage the synthesis of cell-protective prostaglandins.

## Summary

Heavy, long-term alcohol consumption can cause alcoholic liver disease in susceptible people. However, the fact that only a portion—albeit a relatively large one—of heavy drinkers develop alcoholic hepatitis or cirrhosis indicates the importance of heredity, gender, diet, and other forms of liver disease as influences over the risk for alcoholic liver disease. Most of the liver damage caused by alcohol is attributed to alcohol metabolism and the by-products of that metabolism. Liver injury may be caused by direct toxicity of alcohol by-products and also by inflammation that is induced secondarily by these same compounds. Exposure of liver cells to bacterial toxins may be an important contributor to alcoholic liver disease. Progressive liver injury—whether toxic, inflammatory, or both—can lead to fibrosis and, ultimately, to cirrhosis. Increased understanding of the mechanisms of tissue injury has led to innovative treatments for alcoholic liver disease, including the use of corticosteroids, antioxidants, antibiotics, and certain polyunsaturated fats. Ongoing research should provide additional insight into the biological mechanisms underlying liver damage and new approaches to treating the problem in both alcoholic and nonalcoholic patients.

## Footnotes

1 One standard drink is defined as 12 fluid ounces (oz) of beer, 5 fluid oz of wine, or 1.5 fluid oz of distilled spirits.

2 For definitions of this and other technical terms, see glossary, pp. 93–96.

3 An enzyme called catalase also can convert alcohol to acetaldehyde. However, this enzyme rarely plays an important role in humans and will not be discussed further in this article (Lieber 1994).

4 Only abstinent alcoholics are considered candidates for liver transplantation.
